# The Effect of Orlistat on Sterol Metabolism in Obese Patients

**DOI:** 10.3389/fendo.2022.824269

**Published:** 2022-02-23

**Authors:** Yu-Jin Kwon, Go Eun Kwon, Hye Sun Lee, Man Ho Choi, Ji-Won Lee

**Affiliations:** ^1^ Department of Family Medicine, Yongin Severance Hospital, Yonsei University College of Medicine, Yongin, South Korea; ^2^ Molecular Recognition Research Center, Korea Institute of Science and Technology, Seoul, South Korea; ^3^ Biostatistics Collaboration Unit, Department of Research Affairs, Yonsei University College of Medicine, Seoul, South Korea; ^4^ Department of Family Medicine, Gangnam Severance Hospital, Yonsei University College of Medicine, Seoul, South Korea

**Keywords:** obesity, orlistat, sterol, cardiovascular disease, anti-obesity drug

## Abstract

**Background:**

Orlistat, a reversible inhibitor of pancreatic and gastric lipase, is known to have anti-obesity and antioxidant properties. Cholesterol intermediates and metabolites have diverse and important functions in cardiovascular disease. Therefore, we aimed to evaluate the effect of orlistat on sterol metabolism in overweight and obese adults after weight loss during the intervention or weight loss at 12 weeks.

**Methods:**

A total of 51 (27 in the control group and 24 in the experimental group), patients with a BMI of 27 or greater were randomly assigned in a 1:1 ratio to receive either orlistat (120 mg) three times a day plus phentermine hydrochloride (37.5 mg) once daily or a placebo three times a day plus phentermine hydrochloride (37.5 mg) once daily. The primary study outcome was sterol metabolism.

**Results:**

The experimental group exhibited significantly decreased metabolic signatures of serum sterols, free cholesterol, sitosterol, 7α-hydroxycholesterol (7α-OHC), and 7β-OHC at 12 weeks. The experimental group also exhibited significantly decreased metabolic ratios of sitosterol and 7α-OHC to cholesterol at 12 weeks. Regarding changes in sterol signatures from baseline to 6-month follow-up, free cholesterol, plant sterols, and cholesterol precursors tended to decrease with weight loss during the intervention and increase again as the weight was regained in both groups.

**Conclusion:**

Orlistat treatment improves oxysterol metabolism in overweight and obese adults. Our findings support that orlistat plays a crucial role in the process of endothelial dysfunction and atherosclerosis *via* oxysterol modulation.

## Introduction

Obesity, defined as abnormal or excessive fat accumulation, is a crucial risk factor for diabetes, dyslipidemia, nonalcoholic fatty liver disease, and cardiovascular disease (CVD), all of which impair global health (1). Although a healthy lifestyle is the foundation of obesity treatment, lifestyle modification alone produces only modest weight loss that is difficult to sustain. Therefore, adjuvant pharmacotherapy combined with lifestyle treatment could be recommended in patients with a body mass index (BMI) of 30 kg/m^2^ or greater and in those with a BMI of 27 kg/m^2^ or greater with adiposity-related complications (2).

Currently, several anti-obesity drugs are approved and available, but these drugs vary in their efficacy and side-effect profiles (3). Phentermine is a substituted amphetamine and approved for short-term obesity treatment (<12 weeks) (3). However, its cardiac toxicity has not been thoroughly studied, and it is contraindicated in patients with uncontrolled hypertension, coronary artery disease, and stroke due to its sympathomimetic properties. By contrast, orlistat might be preferable in patients with CVD or psychiatric illness or in those who want a drug with a long-term safety record (4). It is a potent and reversible inhibitor of pancreatic and gastric lipase that can decrease fat absorption by as much as 30% (5). Many studies have established that orlistat yields modest weight loss and improves cardiometabolic risk factors (6, 7).

Orlistat has been shown to reduce serum total cholesterol and low-density lipoprotein cholesterol (LDL-C) levels independent of weight loss (6, 8, 9). In line with previous studies, our clinical trial showed that concomitant administration of orlistat and phentermine significantly decreases total cholesterol and non-high-density lipoprotein cholesterol (non-HDL-C) and improves vascular endothelial cell function compared with phentermine alone (10). Therefore, orlistat might decrease CVD risk. Nevertheless, the exact mechanism involved in the previous results is unclear, and further research is needed to evaluate the drug’s long-term benefit on cardiovascular risk.

Although classic lipid parameters are regarded as clinical surrogate markers for CVD, they are limited in explaining atherosclerosis and cardiovascular pathologies (11). Sterols are known to be involved in the pathology of atherosclerosis, neurodegeneration, and inflammation (12). Therefore, the metabolic signatures of sterols, along with the traditional lipid profiles, may provide complementary information to help elucidate the mechanism of chronic inflammatory status, such as obesity and CVD. Patients who were overweight showed low cholesterol absorption efficiency and high cholesterol biosynthesis. However, weight reduction increased the markers of cholesterol absorption and decreased those of cholesterol synthesis in patients with obesity and type 2 diabetics (13).

Oxysterols are enriched in pathologic structures, such as macrophage foam cells and atherosclerotic lesions. Patients with acute coronary syndrome and stable angina had higher serum oxysterol concentration and a higher metabolic ratio of lathosterol to cholesterol and lanosterol to cholesterol than healthy individuals (14, 15). After statin therapy, significant decreases in cholesterol precursors were found in patients with stable angina and acute coronary syndrome (16). However, few studies have evaluated the effect of weight reduction with an anti-obesity drug on sterol metabolism. Furthermore, to our knowledge, no data currently exists that show the changes in sterol metabolism with weight regain after treatment discontinuation. Therefore, we aimed to evaluate the effect of orlistat on sterol metabolism in overweight and obese adults. Additionally, we examined the changes in various sterol signatures after weight loss and subsequent weight regain with the post-trial follow-up.

## Methods

### Randomized Trial

Detailed information about the study’s method and recruitment of patients was described in our previous article (10). The original study was a randomized, double-blind, placebo-controlled 12-week clinical trial (ClinicalTrials.gov No., NCT03675191).

In brief, 113 patients who were obese (BMI ≥ 30 kg/m^2^) or overweight (BMI ≥ 27 kg/m^2^) with at least one weight-related complication, aged 20–70 years were enrolled in the trial between October 2018 and May 2019 at Yongin Severance Hospital (Yongin, Korea). Participants were randomly assigned in a 1:1 ratio to receive orlistat (120 mg) three times a day plus phentermine hydrochloride (37.5 mg) once daily or a placebo three times a day plus phentermine hydrochloride (37.5 mg) once daily for 12 weeks. The experimental group (the orlistat group) included 57 participants who received orlistat plus phentermine. The control group included 56 participants who received placebo plus phentermine. The last visits for the trial were completed in May 2019 after the 12-week intervention. This study was completed by 50 of the 57 participants in the experimental group and 46 of the 56 participants in the control group. At the end of 12 weeks, all participants discontinued the intervention and returned to their usual daily life without any treatment.

### Post-Trial Follow-Up

**Figure 1:**
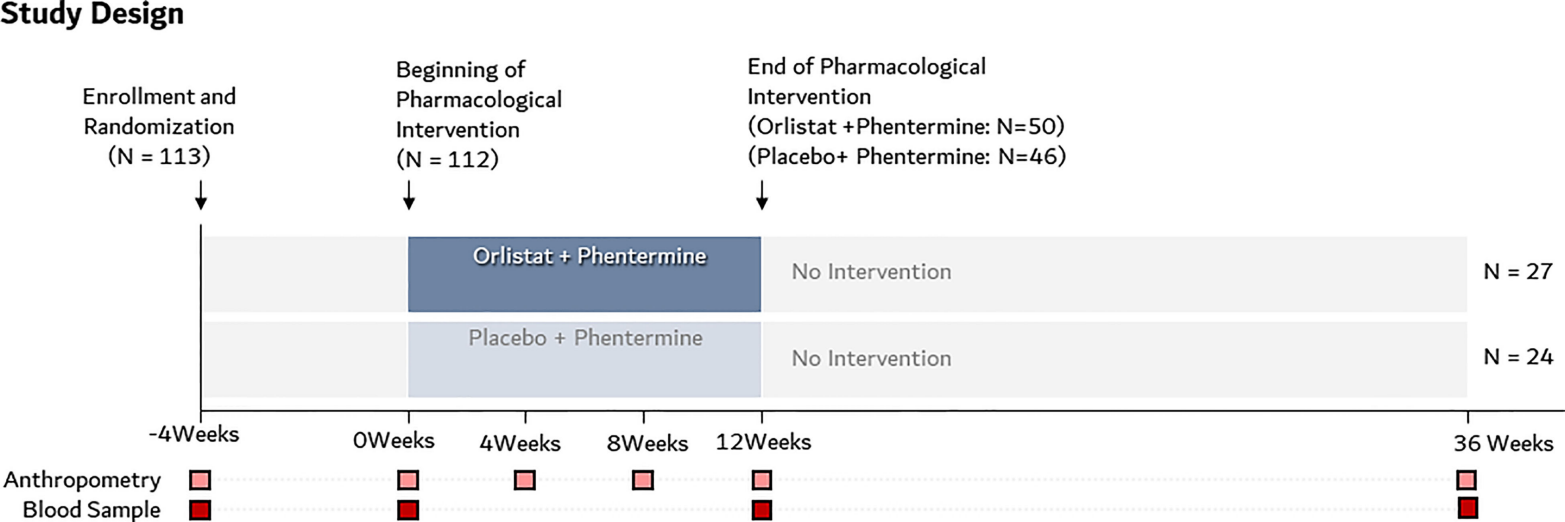
Study design schematic of the intervention and follow-up period.

Six months after completion of the final trial visits, patients were invited to participate in the follow-up study *via* telephone and 51 of 96 (53%) agreed to participate in this post-trial follow-up. Overall, 27 of the 50 patients who were assigned to control group and 24 of the 46 assigned to experimental group were enrolled in this follow-up study. The study visits occurred between June 2019 and October 2019. The protocol was approved by the institutional review boards of Yongin Severance Hospital (IRB No. 9-2019-0003). At the post-trial visit, each participant provided written informed consent. This study was performed in compliance with the Declaration of Helsinki. Enrolled patients underwent blood sampling and measurements of height, weight, waist circumference, body composition, and blood pressure. The scheme of study was illustrated in **Figure 1**.

### Trial Outcomes

Body weight, waist circumference, systolic blood pressure, and diastolic blood pressure were measured at screening, baseline, and 4, 8, 12, and 6-month follow-up. BMI was a person’s weight in kilograms divided by the square of height in meters. Waist circumference was measured using a measuring tape on the horizontal plane midway between the lowest rib and the iliac crest. Laboratory tests and blood sampling were measured at baseline, 12 weeks, and 6-month follow-up. Health-related (physical activity, smoking, alcohol consumption) questionnaires were administered at baseline, 12 weeks, and 6-month follow-up. Participants were categorized into never smoker, ex-smoker, and current smoker groups. An alcohol drinker was defined as a person who drinks alcohol more than once a month. Physical activity was defined as undertaking light to moderate exercise more than two times per week. We used a binary variable with the presence or absence of history of hypertension, dyslipidemia, or diabetes, according to answers from a self-reported questionnaire. Blood samples were collected after more than eight hours of fasting. Lipids (total cholesterol, LDL-C, non-HDL-C, triglycerides, HDL-C) were analyzed using the enzymatic color test. The primary efficacy endpoint was change in sterols from baseline to 12 weeks. The secondary endpoints were changes in sterols from 12 weeks to the 6-month follow-up.

### Quantitative Profiling of Serum Sterols

Serum levels of endogenous sterols and plant sterols were measured as described previously (16). Briefly, 20 μL serum samples spiked with 20 μL of a mixture of deuterium-labeled internal standards were added to 0.5 mL methanol and shaken using TissueLyser (Qiagen; Hilden, Germany) at 25 Hz for 1 min. After centrifugation at 12,000 rpm for 10 min, the supernatant was extracted using an H-PPT cartridge, and the eluate was evaporated under N_2_ gas at 40°C followed by drying in a vacuum desiccator at least 30 min. The dried extract was trimethylsilyl derivatized, and an aliquot (2 μL) was then injected into the gas chromatography-mass spectrometry (GC-MS) system.

### Statistical Analysis

Differences in baseline characteristics between the control and experimental groups were compared using the independent *t*-test for continuous variables and the chi-square test for categorical variables. Differences after intervention within groups were compared using the independent two sample t-test. Differences after intervention within groups were also compared using the analysis of covariance after adjusting for age, sex, and baseline body weight.

A linear mixed model for a repeated-measures covariance pattern model with unstructured covariance within participants was used to assess the longitudinal associations between the groups and subsequent changes in clinical variables and sterols over time. The outcomes were repeated measures of clinical variables and sterols from baseline to 12 weeks and 6-month follow-up. The model included group, time, and group × time interaction as fixed effects. All the *post hoc* comparisons were analyzed using the Bonferroni method.

We also checked the results using the nonparametric test statistical method (data not shown). The results from the two methods were similar. Significance tests were two sided, with an alpha value of 0.05. All statistical analyses were performed using SAS version 9.4 (SAS Institute Inc., Cary, NC, USA).

## Results

### Clinical Characteristics

Of the 96 patients who completed the original clinical trial, 51 patients (43 women and 8 men) participated in the post-trial follow-up. Of these patients, 27 had been assigned to the control group and 24 had been assigned to the experimental group. The mean (min, max) age of total participants were 48 (20–70) years old. The baseline characteristics of these participants who participated in the 6-month follow-up are presented in **Supplementary Material: Appendix Table 1**. The two groups of follow-up participants had similar demographic and clinical characteristics at baseline. The mean ages were 46.4 ± 12.8 years in the Experimental group and 49.5 ± 10.6 years in the control group. The mean body weight values were 80.5 ± 14.7 kg in the experimental group and 83.1 ± 18.5 kg in the control group.

### Changes in Body Composition

In both groups, BMIs, fat mass, and fat percentage decreased during the 12-week intervention (**Table 1**) but increased at the 6-month follow-up visit (**Supplementary Material: Appendix Table 2**). The changes in body composition with time were significantly greater in the experimental group (**Table 1**).

**Table 1:** Baseline characteristics and Changes at 12 weeks in overweight or obese participants treated with placebo plus phentermine or orlistat plus phentermine.

Variables	Placebo + Phentermine (N = 27) (Control group)			Orlistat + Phentermine (N = 24) (Experimental group)			p^b^
	Baseline	12 Weeks	p^a^	Baseline	12 Weeks	p^a^	
Weight	83.1 ± 18.5	76.9 ± 17.1	<0.001	80.5 ± 14.7	72.6 ± 13.2	<0.001	
* Change*		−6.2 ± 3.0			−7.9 ± 3.2		0.054
Change*		−6.9 (0.6)			−8.6 (0.8)		0.040
BMI	31.3 ± 4.8	29.0 ± 4.9	<0.001	30.9 ± 3.5	27.7 ± 3.2	<0.001	
* Change*		−2.3 ± 0.9			−3.2 ± 1.2		0.002
Change*		−2.4 (0.2)			−3.2 (0.3)		0.003
WC	103.5 ± 10.1	94.4 ± 11.0	<0.001	102.2 ± 9.7	90.2 ± 9.8	<0.001	
* Change*		−9.1 ± 5.1			−12.0 ± 6.8		0.091
Change*		−9.9 (1.2)			−11.8 (1.5)		0.202
Muscle mass	27.6 ± 6.8	26.4 ± 6.3	<0.001	25.6 ± 6.4	24.5 ± 5.8	<0.001	
* Change*		−1.2 ± 1.0			−1.1 ± 0.9		0.556
Change*		−1.1 (0.2)			−1.1 (0.2)		0.762
Fat mass	33.1 ± 9.5	29.1 ± 9.4	<0.001	34.0 ± 7.1	27.9 ± 6.4	<0.001	
* Change*		−4.0 ± 2.3			−6.1 ± 2.9		
Change*		−4.1 (0.6)			−6.2 (0.7)		0.006
Fat, %	39.7 ± 5.7	37.5 ± 6.8	<0.001	42.3 ± 5.4	38.4 ± 5.3	<0.001	
* Change*		−2.2 ± 2.1			−3.9 ± 2.2		0.007
Change*		−2.6 (0.5)			−4.2 (0.6)		0.014
SBP	133.6 ± 14.2	120.2 ± 13.6	<0.001	126.7 ± 15.3	114.6 ± 11.3	<0.001	
* Change*		−13.4 ± 11.8			−12.0 ± 11.3		0.684
Change*		−16.3 (2.7)			−16.8 (3.2)		0.886
DBP	88.0 ± 13.4	77.0 ± 10.6	<0.001	84.6 ± 12.4	73.0 ± 9.8	<0.001	
* Change*		−11.0 ± 9.7			−11.6 ± 9.3		0.817
Change*		−12.0 (2.3)			−13.6 (2.7)		0.546
Glucose	105.0 ± 13.5	103.1 ± 11.7	0.479	118.2 ± 69.4	104.1 ± 11.1	0.297	
* Change*		−1.9 ± 13.7			−14.0 ± 64.5		0.374
* Change*		3.8 (11.0)			−5.3 (13.0)		0.500
Cholesterol	199.3 ± 39.0	181.8 ± 35.6	0.004	197.5 ± 30.5	170.1 ± 26.6	<0.001	
* Change*		−17.4 ± 28.4			−27.4 ± 18.9		0.153
Change*		−23.6 (5.8)			−36.0 (6.8)		0.084
Triglyceride	150.5 ± 73.8	113.6 ± 53.0	0.002	124.5 ± 56.3	108.3 ± 51.5	0.061	
* Change*		−36.9 ± 54.9			−16.3 ± 40.4		0.138
Change*		−38.4 (11.8)			−21.8 (14.0)		0.254
HDL-C	51.7 ± 10.0	49.5 ± 9.9	0.054	55.6 ± 12.4	49.2 ± 9.6	0.001	
* Change*		−2.3 ± 5.8			−6.4 ± 6.9		0.024
Change*		−2.5 (1.6)			−6.8 (1.9)		0.027
LDL-C	126.4 ± 31.4	115.0 ± 28.2	0.019	122.1 ± 23.7	106.3 ± 20.8	<0.001	
* Change*		−11.4 ± 23.7			−15.8 ± 12.2		0.408
Change*		−16.5 (4.6)			−22.4 (5.4)		0.295

BMI, body mass index; DBP, diastolic blood pressure; HDL-C, high-density lipoprotein cholesterol; LDL-C, low-density lipoprotein cholesterol; SBP, systolic blood pressure; TC, total cholesterol; WC, waist circumference.

Data are presented mean ± standard deviations or estimated mean (SE).

a Calculated using the paired t-test.

b Comparison of in-group changes; Calculated using the independent two sample t-test.

Change * Comparison of in-group changes; Calculated using the analysis of covariance after adjusting for age, sex and baseline body weight.

### Changes in Sterols During the 12-Week Intervention and the Post-Trial Follow-Up

After the weight loss intervention, free cholesterol was significantly decreased in the Experimental group but not in the Control group. Most plant sterols decreased in both groups; however, only sitosterol significantly decreased in the Experimental group compared with the Control group. In both groups, cholesterol esters (Chol-M, Chol-P, and Chol-A) were increased, whereas a cholesterol precursor, desmosterol, was decreased. However, no significant differences were found between the two groups. We found that 7α-hydroxycholesterol (7α-OHC) and 7β-hydroxycholesterol (7β-OHC) were significantly decreased in the Experimental group after adjusting for age, sex, and baseline body weight. The changes in these two oxysterol levels were greater in the experimental group than in the control group (p = 0.032; p = 0.030; **Table 2**). The results were similar with those from unadjusted model.

**Table 2:** Individual sterols Changes in overweight or obese participants treated with placebo plus phentermine or orlistat plus phentermine for 12 weeks.

Sterols, μg/mL	Placebo + Phentermine (N = 27) (Control group)			Orlistat + Phentermine (N = 24) (Experimental group)			
	Baseline	12 Weeks	p^a^	Baseline	12 Weeks	p^a^	p^b^
Cholesterol	643.5 ± 130.4	641.8 ± 148.9	0.937	653.8 ± 132.7	592.0 ± 129.5	0.010	
* Change*		−1.7 ± 112.6			−61.7 ± 108.2		0.059
Change*		−18.6 (26.9)			−87.7 (31.7)		0.039
Sitosterol	0.54 ± 0.23	0.45 ± 0.21	0.071	0.65 ± 0.46	0.36 ± 0.13	0.002	
* Change*		−0.09 ± 0.24			−0.29 ± 0.40		0.037
Change*		−0.19 (0.08)			−0.43 (0.09)		0.012
Campesterol	0.81 ± 0.40	0.60 ± 0.29	0.004	0.89 ± 0.67	0.49 ± 0.19	0.001	
* Change*		−0.21 ± 0.34			−0.40 ± 0.53		0.130
Change*		−0.35 (0.10)			−0.59 (0.12)		0.063
Stigmasterol	0.13 ± 0.02	0.10 ± 0.02	<0.001	0.13 ± 0.03	0.09 ± 0.02	<0.001	
* Change*		−0.02 ± 0.03			−0.04 ± 0.03		0.105
Change*		−0.03 (0.01)			−0.03 (0.01)		0.059
Chol-M	37.1 ± 22.1	96.4 ± 76.2	<0.001	41.9 ± 27.99	85.19 ± 64.65	0.006	
* Change*		59.3 ± 62.7			43.3 ± 69.9		0.393
Change*		52.0 (15.9)			41.7 (18.4)		0.592
Chol-P	380.1 ± 91.4	532.3 ± 212.0	0.004	399.9 ± 145.5	494.1 ± 216.9	0.068	
* Change*		152.2 ± 196.9			94.2 ± 241.3		0.350
Change*		94.5 (46.9)			50.1 (58.5)		0.467
Chol-A	329.0 ± 184.8	1138.2 ± 820.7	<0.001	349.3 ± 105.6	845.9 ± 575.3	0.001	
* Change*		809.3 ± 788.2			496.5 ± 620.0		0.125
Change*		622.9 (164.6)			340.8 (194.1)		0.164
Desmosterol	62.9 ± 15.1	54.4 ± 13.9	0.012	60.4 ± 12.0	50.1 ± 12.6	0.002	
* Change*		−8.6 ± 16.5			−10.3 ± 11.4		0.667
Change*		−12.9 (3.5)			−16.0 (4.0)		0.462
DHC	84.9 ± 27.8	76.3 ± 19.1	0.135	82.0 ± 16.0	72.6 ± 12.9	0.03	
* Change*		−8.6 ± 28.9			−9.3 ± 19.2		0.914
Change*		−10.8 (6.0)			−10.3 (7.0)		0.945
Lathosterol	461.5 ± 256.2	368.1 ± 238.6	0.011	393.2 ± 206.3	308.8 ± 161.2	0.065	
* Change*		−93.4 ± 177.1			−84.4 ± 213.0		0.870
Change*		−101.6 (47.8)			−99.5 (56.4)		0.972
Lanosterol	84.0 ± 53.6	78.3 ± 54.8	0.448	84.5 ± 45.9	68.2 ± 34.0	0.027	
* Change*		−5.7 ± 38.6			−16.3 ± 33.8		0.307
Change*		−14.3 (8.6)			−23.8 (10.1)		0.363
7α-OHC	36.2 ± 7.0	33.5 ± 25.3	0.621	57.4 ± 56.1	28.3 ± 9.5	0.008	
* Change*		−2.6 ± 27.5			−29.1 ± 49.4		0.026
Change*		−4.6 (9.7)			−30.7 (14.5)		0.032
7β-OHC	17.1 ± 1.6	16.6 ± 4.6	0.641	20.2 ± 6.7	16.0 ± 2.0	0.002	
* Change*		−0.5 ± 5.0			−4.2 ± 5.7		0.017
Change*		−0.8 (1.3)			−4.5 (1.6)		0.03
Ketosterol	23.2 ± 6.0	17.6 ± 3.5	<0.001	56.6 ± 124.4	18.4 ± 5.8	0.189	
* Change*		−5.6 ± 4.9			−38.2 ± 120.1		0.196
Change*		−7.8 (20.3)			−37.9 (23.9)		0.227
27-OHC	12.4 ± 2.6	13.0 ± 3.7	0.503	12.6 ± 3.3	11.7 ± 3.7	0.500	
* Change*		0.6 ± 4.2			−0.9 ± 4.2		0.224
Change*		0.2 (1.0)			−1.7 (1.2)		0.121
24-OHC	10.9 ± 4.3	13.5 ± 7.8	0.109	10.8 ± 5.3	10.7 ± 6.8	0.885	
* Change*		2.6 ± 8.2			−0.1 ± 7.6		0.221
Change*		3.04 (2.0)			0.21 (2.32)		0.241

7α-OHC, 7α-hydroxycholesterol; 7β-OHC, 7β-hydroxycholesterol; 24-OHC, 24-oxysterol; 27-OHC, 27-oxysterol; Chol-M, cholesteryl myristate; Chol-A, cholesteryl arachidonate; Chol-P, cholesteryl palmitate; DHC, 7-dehydrocholesterol; Ketosterol, 7-ketocholesterol.

Data are presented mean ± standard deviations or estimated mean (SE).

a Calculated using the paired t-test.

b Comparison of in-group changes; Calculated using the independent two sample t-test.

Change * Comparison of in-group changes; Calculated using the analysis of covariance after adjusting for age, sex and baseline body weight.

Changes in metabolic ratios of sterols were observed between the Control group and the Experimental group at baseline and 12 weeks (**Table 3**). These metabolic alterations against cholesterol were similar to those of respective sterols except sitosterol and 7α-OHC to cholesterol ratios. Both sitosterol and 7α-OHC to cholesterol ratios were significantly decreased in the Experimental group.

**Table 3:** Changes in metabolic ratio of the sterols in overweight or obese participants treated with placebo plus phentermine or orlistat plus phentermine for 12 weeks.

Placebo + Phentermine (N = 27) (Control group)				Orlistat + Phentermine (N = 24) (Experimental group)			
Variables	Baseline	12 Weeks	p^a^	Baseline	12 Weeks	p^a^	p^b^
Desmo/Chol	0.098 ± 0.016 0.087 ± 0.021		0.021	0.094 ± 0.020 0.087 ± 0.022		0.079	
* Change*	−0.012 ± 0.025			−0.008 ± 0.020			0.529
Change*	−0.016 (0.005)			−0.013 (0.006)			0.620
DHC/Chol	0.133 ± 0.039 0.122 ± 0.028		0.249	0.127 ± 0.023 0.127 ± 0.027		0.896	
* Change*	−0.011 ± 0.049			−0.001 ± 0.026			0.345
Change*	−0.011 (0.009)			0.003 (0.011)			0.219
Latho/Chol	705.7 ± 377.1	571.8 ± 339.4	0.026	613.5 ± 338.1	517.8 ± 228.5	0.147	
* Change*	−133.9 ± 293.7			−95.7 ± 312.1			0.655
Change*	−126.3 (73.2)			−93.1 (86.5)			0.711
Lano/Chol	126.2 ± 75.6	120.5 ± 77.1	0.552	133.3 ± 77.9	118.2 ± 56.6	0.149	
* Change*	−5.7 ± 49.6			−15.2 ± 49.7			0.502
Change*	−13.3 (11.8)			−20.5 (13.9)			0.619
Sito/Chol	0.84 ± 0.31	0.71 ± 0.34	0.078	0.99 ± 0.67	0.64 ± 0.32	0.001	
* Change*	−0.13 ± 0.38			−0.35 ± 0.46			0.071
Change*	−0.25 (0.10)			−0.50 (0.12)			0.037
Camp/Chol	1.26 ± 0.52	0.95 ± 0.45	0.007	1.36 ± 1.00	0.86 ± 0.49	0.004	
* Change*	−0.31 ± 0.56			−0.50 ± 0.60			0.245
Change*	−0.40 (0.14)			−0.67 (0.16)			0.206
Stigma/Chol	0.20 ± 0.04	0.16 ± 0.05	0.001	0.20 ± 0.05	0.17 ± 0.06	<0.001	
* Change*	−0.04 ± 0.04			−0.04 ± 0.04			0.826
Change*	−0.05 (0.01)			−0.05 (0.01)			0.884
Latho/Camp	764.4 ± 709.2	754.2 ± 660.2	0.878	551.5 ± 340.0	717.4 ± 391.6	0.020	
* Change*	−10.2 ± 341.7			165.9 ± 326.0			0.067
Change*	−2.6 (82.8)			169.7 (97.6)			0.093
7α-OHC/chol	57.3 ± 11.0	54.5 ± 43.3	0.743	85.8 ± 7.1	47.9 ± 11.2	0.010	
* Change*	−2.8 ± 44.0			−37.8 ± 66.2			0.034
Change*	−3.3 (13.7)			−37.3 (16.2)			0.047
7β-OHC/chol	27.3 ± 4.8	27.2 ± 9.7	0.915	31.3 ± 9.1	27.8 ± 4.7	0.089	
* Change*	−0.2 ± 8.4			−3.4 ± 9.4			0.201
Change*	0.1 (2.2)			−2.6 (2.6)			0.309
27-OHC/chol	19.8 ± 4.7	20.3 ± 5.0	0.624	19.6 ± 4.5	20.5 ± 6.9	0.489	
* Change*	0.6 ± 6.0			0.9 ± 6.3			0.844
Change*	1.0 (1.5)			1.0 (1.8)			0.999
24-OHC/chol	17.2 ± 6.8	20.9 ± 11.7	0.152	16.5 ± 7.6	17.7 ± 10.0	0.644	
* Change*		3.7 ± 13.1			1.1 ± 11.3		0.449
Change*	4.9 (3.0)			2.6 (3.6)			0.528

7α-OHC, 7α-hydroxycholesterol; 7β-OHC, 7β-hydroxycholesterol; 27-OHC, 27-Oxysterol; 24-OHC, 24-Oxysterol; Camp, campesterol; Chol, cholesterol; Desmo, desmosterol; DHC, 7-dehydrocholesterol; Lano, lanosterol; Latho, lathosterol; Stigma, stigmasterol.

Data are presented mean ± standard deviations or estimated mean (SE).

a Calculated using the paired t-test.

b Comparison of in-group changes; Calculated using the independent two sample t-test.

Change * Comparison of in-group changes; Calculated using the analysis of covariance after adjusting for age, sex and baseline body weight.

**Figure 2:**
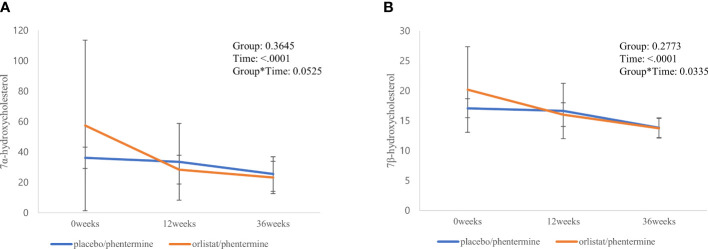
Significant changes in individual sterols and their metabolic ratios from baseline through the follow-up period. **(A)** 7α-hydroxycholesterol. **(B)** 7β-hydroxycholesterol.

**Figure 2** illustrates the linear mixed model of the two oxysterols (7α-OHC and 7β-OHC) between the two groups during the intervention and post-trial follow-up period. Both groups maintained continuously decreasing trends for 7α-OHC and 7β-OHC levels until the follow-up period after weight loss. The decreased changes in 7α-OHC and 7β-OHC levels in the Experimental group remained greater than in the Control group during the intervention and post-trial follow-up period. Additionally, there was a significant time by group interaction in 7β-OHC levels (p = 0.034) and a borderline interaction in 7α-OHC levels between the two groups (p = 0.053).

Changes in various sterols and their metabolic ratios from baseline through the follow-up period are presented in **Supplementary Material: Appendix Table 3**. In both groups, free cholesterol, plant sterols, and cholesterol precursors showed a tendency to decrease and then increase again as weight regain began after the weight loss intervention. However, cholesteryl esters maintained continuous increasing trends until the follow-up period after the weight loss intervention. Additionally, a borderline interaction in 7α-OHC/cholesterol levels was identified between the two groups during the intervention and post-trial follow-up period.

## Discussion

We found that the metabolic signatures of serum sterols, free cholesterol, sitosterol, 7α-OHC, and 7β-OHC were significantly decreased in adults who received orlistat plus phentermine after the weight loss intervention. Furthermore, cholesteryl esters maintained a continuous increasing trend across the intervention and follow-up period, whereas 7α-OHC and 7β-OHC levels exhibited decreasing trends until the follow-up period after weight loss in both groups.

Obesity is associated with chronic oxidative stress and inflammation, which initiates the progression of atherosclerosis (17). Orlistat, a reversible inhibitor of intestinal lipases, has favorable effects on weight loss and some cardiometabolic parameters (6, 7). Additionally, orlistat has been shown to inhibit NF-κB-mediated inflammation and improve endothelial dysfunction (18–21). Hence, orlistat could be useful to ameliorate the progression of atherosclerosis in obesity. The disorder of lipid metabolism plays a pivotal role in the development of atherosclerosis (22). Although classical lipid profiles are used to estimate coronary artery disease risk, impairments in sterol homeostasis manipulated with free cholesterol and its precursors and metabolites are known to be powerful predictors of developing cardiovascular events, even in the early stages of atherosclerosis (23, 24).

Oxysterols, oxidized metabolites of cholesterol, are present in mammalian tissues at very low concentrations. However, they are enriched in pathologic structures such as macrophage foam cells, atherosclerotic lesions, and the blood of hypercholesterolemic individuals (14, 15). Previous studies have identified the cytotoxic and pro-apoptotic activities of oxysterols, leading to the presumption that they also possess pro-atherosclerotic properties (25–27). Oxysterols diffuse much better through phospholipid membranes than cholesterol especially because the half-life for 7α-OHC and 7β-OHC exchange between lipid vesicles is 100 times higher than that observed for cholesterol (28, 29). 7α-OHC and 7β-OHC are known to the most abundant forms in the arterial compartment (27) and were demonstrated to induce an inflammatory phenotype in human endothelial cells (30).

In our study, overweight or obese adults treated with orlistat plus phentermine had significantly lower 7α-OHC and 7β-OHC levels, as well as a significantly lower metabolic ratio of 7α-OHC to cholesterol, than those treated with phentermine alone after the weight loss intervention even in the absence of different changes in serum total cholesterol, LDL-C, and triglycerides. Furthermore, the decreasing change in 7β-OHC levels was more prominent in adults who received orlistat plus phentermine than those who received phentermine alone until the post-trial follow-up period. Our results suggest that orlistat might play a role in the process of endothelial dysfunction and atherosclerosis *via* oxysterol modulation. In our previous study (10), orlistat improved endothelial-dependent flow-mediated vasodilation in obese adults independent of weight loss, which supports the current findings. However, the mechanism by which orlistat changes oxysterol levels is still unknown, so additional mechanism studies are necessary. The values of 7α-OHC and 7β-OHC between the two groups with the placebo/orlistat and orlistat/phentermine are approximately the same at 36 weeks. During the 6months follow-up period, both groups regained the weight (see **Supplementary Material: Appendix Table 2**). The estimated mean (standard errors) of body weight values were 78.9 (3.0) kg in the experimental group and 75.0 (3.2) kg in the control group. The weight regain may increase the metabolic activity of cholesterol 7α-hydroxylase (31, 32).

Lecithin–cholesterol acyltransferase (LCAT), which esterifies free cholesterol, has been proposed to play antiatherogenic properties (33). Increased cholesteryl esters by LCAT reduces diet-induced atherosclerosis in scavenger receptor class B member I knockout mice. By contrast, inhibition of the intracellular esterification of cholesterol by acyl-CoA:cholesterol acyltransferase inhibitors accelerate atherosclerosis and increases cardiovascular events in human clinical trials (34, 35). Although the exact mechanism involved in our results is unclear, we found cholesteryl esters increased after weight loss intervention and maintained a continuous increasing trend until the follow-up period in both groups. Further investigations are required to identify the relationship between weight loss and anti-obesity treatments on cholesteryl ester production.

Adults with obesity are known to have increased serum concentrations of cholesterol precursors, which reflect the biosynthesis of endogenous cholesterol, but decreased levels of plant sterols, known as markers of cholesterol absorption (35–37). In addition, weight reduction decreases cholesterol precursors, such as lanosterol, lathosterol, and desmosterol, and increases plant sterols, such as sitosterol and campesterol (13, 35–37). Our data are consistent with previous studies, showing that free cholesterol and cholesterol precursors tended to decrease with weight loss and increase again as the weight was regained in both groups. However, plant sterols also showed this same pattern. Inconsistencies with previous studies might be due to differences in the characteristics of the study population, a different diet control and the use of anti-obesity medications, or a different degree weight loss and duration of the intervention in this trial. Interestingly, unlike other plant sterols, sitosterol and its ratio to cholesterol were significantly decreased after weight loss in adults who received orlistat plus phentermine. Although the exact reason remains unknown, sitosterol is most effectively returned to the gut among all plant sterols, thereby resulting in the lowest absorption rate (38). Another possible explanation for these results is that orlistat limits dietary cholesterol absorption by the inhibition of Niemann-Pick C1-like 1 (NPC1L1) transport protein, as well as by the inhibition of intestinal lipase (39). The NPC1L1 was proposed to play an important role in the competitive uptake of plantar sterols and cholesterol across the enterocytes’ apical membrane (40).

### Limitations and Strengths

Our study has several limitations. First, this study combined data from a clinical trial with data from a post-trial observational follow-up study. Although randomized clinical trials (RCT) are considered the gold standard for producing reliable evidence, they are time consuming and expensive. By contrast, observational studies run the risk of containing confounding biases by nature, wherein effects are examined in real world settings without interference. Therefore, the extension of a clinical trial with an observational period may provide valid and reliable real-world evidence. Second, it was difficult to see the effect of orlistat alone in our RCT because both study arms received phentermine. Future studies are needed to determine the effect of orlistat alone on sterol metabolisms. Third, we collected no information on dietary cholesterol or plant sterols in our 24-h dietary recall. It is also possible that self-controlled caloric restriction does not adequately reflect cholesterol absorption, biosynthesis, and excretion. Fourth, only approximately 50% participants who enrolled original RCT participate the post follow-up trial. There could be selection bias.

## Conclusions

Orlistat treatment improved oxysterol metabolism in overweight and obese adults, and the favorable changes in oxysterol were maintained until 6 months after orlistat treatment ended. Thus, orlistat may have pivotal role in the process of endothelial dysfunction and atherosclerosis *via* oxysterol modulation. Additionally, adults treated with orlistat had significantly decreased free cholesterol, sitosterol, and metabolic ratios of sitosterol after weight loss, suggesting orlistat as another therapeutic option for hypercholesterolemia. Further investigations are required to identify the role of orlistat in various sterol signatures and metabolisms.

## Data Availability Statement

The raw data supporting the conclusions of this article will be made available by the authors, without undue reservation.

## Ethics Statement

The studies involving human participants were reviewed and approved by Institutional review boards of Yongin Severance Hospital. The patients/participants provided their written informed consent to participate in this study.

## Author Contributions

Concept and design: Y-JK, J-WL, and MC. Performed experiment: GK and MC. Acquisition, analysis, or interpretation of data: Y-JK, GK, J-WL, and MC. Drafting of the manuscript: Y-JK, GK, J-WL, and MC. Critical revision of the manuscript for important intellectual content: Y-JK, GK, J-WL, and MC. Statistical analysis: Y-JK, GK, J-WL, and MC. Obtained funding: J-WL. Administrative, technical, or material support: GK and MC. Supervision: J-WL and MC. All authors contributed to the article and approved the submitted version.

## Funding

This study was supported by a research grant (No. KSSO202006) from the Korean Society for the Study of Obesity. This work was supported by the Technology Innovation Program (20002781, A Platform for Prediction and Management of Health Risk Based on Personal Big Data and Lifelogging) funded by the Ministry of Trade, Industry and Energy, Korea, to J-WL. The funding sources had no role in the design and conduct of the study; collection, management, analysis, and interpretation of the data; preparation, review, or approval of the manuscript; and decision to submit the manuscript for publication.

## Conflict of Interest

The authors declare that the research was conducted in the absence of any commercial or financial relationships that could be construed as a potential conflict of interest.

## Publisher’s Note

All claims expressed in this article are solely those of the authors and do not necessarily represent those of their affiliated organizations, or those of the publisher, the editors and the reviewers. Any product that may be evaluated in this article, or claim that may be made by its manufacturer, is not guaranteed or endorsed by the publisher.
