# Supplementary material for: The Effect of Orlistat on Sterol Metabolism in Obese Patients
Source: Front Endocrinol (Lausanne). 2022 Feb 23;13:824269. doi: 10.3389/fendo.2022.824269 (PMC8905288; doi:10.3389/fendo.2022.824269)
Supplement: Supplementary file 1 [file DataSheet_1.docx]

**Appendix Table 1. Baseline characteristics of the study population**

|  | Placebo + phentermine  (P group) | Orlistat + phentermine  (OP group) | p-value |
| --- | --- | --- | --- |
| N | 27 | 24 |  |
| Variables |  |  |  |
| Age, years | 50 (43-57) | 47 (39-56) | 0.428 |
| Sex (women), n (%) | 21 (77.8) | 22 (91.7) | 0.255 |
| Weight, kg | 76.9 (72.3-87.6) | 75.0 (71.7-89.0) | 0.521 |
| BMI, kg/m^2^ | 29.8 (27.5-33.6) | 30.2 (27.8-33.0) | 0.962 |
| Waist circumference, cm | 102 (96.5-108) | 100 (93.5-112.3) | 0.461 |
| Smokers(yes), n (%) | 5 (19.2) | 2 (8.3) | 0.485 |
| Alcohol drinkers (yes), n (%) | 12 (46.2) | 13 (56.5) | 0.469 |
| Physical activity (yes), n (%) | 5 (19.2) | 5 (20.8) | >0.999 |
| Systolic blood pressure, mmHg | 138 (124-142) | 125.5 (120-130) | 0.049 |
| Diastolic blood pressure, mmHg | 89 (75-100) | 85 (77.5-90) | 0.427 |
| Glucose, mg/dl | 102 (98-110) | 98 (95-109) | 0.416 |
| Total cholesterol, mg/dl | 192 (177-222) | 195.5 (177-210) | 0.985 |
| LDL cholesterol, mg/dl | 124 (111-141) | 121.5 (114-132) | 0.720 |
| HDL cholesterol, mg/dl | 48 (44-61) | 52.5 (45.5-61.5) | 0.374 |
| Triglycerides, mg/dl | 139 (93-175) | 115 (82-143) | 0.186 |
| Sterols, μg/mL |  |  |  |
| Cholesterol | 636.0 (533.8-726.5) | 631.3 (561.6-759.4) | 0.850 |
| Sitosterol | 0.50 (0.38-0.69) | 0.53 (0.39-0.71) | 0.546 |
| Campesterol | 0.72 (0.56-1.03) | 0.73 (0.57-1.02) | 0.902 |
| Stigmasterol | 0.12 (0.11-0.14) | 0.12 (0.12-0.14) | 0.716 |
| Chol_M | 35.3 (22.6-43.8) | 32.4 (22.3-49.4) | 0.777 |
| Chol_P | 379.9 (319.1-439.2) | 387.1 (325.3-442.5) | 0.865 |
| Chol_A | 278.6 (228.5-369.9) | 302.7 (234.6-436.4) | 0.485 |
| Desmosterol | 60.1 (53.4-71.5) | 57.2 (52.3-71.5) | 0.571 |
| DHC | 80.3 (68.2-97.0) | 85.1 (70.9-92.8) | 0.664 |
| Lathosterol | 446.8 (225.2-596.1) | 369.3 (228.3-506.9) | 0.308 |
| Lanosterol | 67.5 (43.6-110.5) | 74.3 (55.2-106.9) | 0.706 |
| 7α-OHC | 36.7 (32.1-41.2) | 44.4 (31.7-57.9) | 0.059 |
| 7β-OHC | 17.2 (15.9-18.4) | 18.1 (15.8-21.2) | 0.095 |
| Ketosterol | 22.8 (17.6-27.3) | 26.1 (19.7-42.8) | 0.081 |
| 27-OHC | 12.0 (10.5-14.2) | 11.5 (9.8-14.2) | 0.925 |
| 24-OHC | 10.5 (9.8-14.2) | 11.7 (5.1-14.5) | 0.799 |

BMI, body mass index; LDL, low-density lipoprotein; HDL, high-density lipoprotein.

Data are presented as median (IQR) for continuous variables or number (%) for categorical variables.

P-values were calculated using the Mann-Whitney U test.

**Appendix Table 2. Changes in clinical variables from baseline through the follow-up period**

| Variables | Time | Placebo + Phentermine | Orlistat + Phentermine | Overall p-value^a^ |
| --- | --- | --- | --- | --- |
|  |  |  |  |  |
|  |  | Estimated means (SE) | Estimated means (SE) |  |
| Weight, kg | 0 Weeks | 83.1 (3.2) | 80.5 (3.4) | group: 0.425  time: <.001  group*time: 0.084 |
|  | 12 Weeks | 76.9 (3.0) | 72.6 (3.1) |  |
|  | 36 Weeks | 78.6 (3.0) | 75.0 (3.2) |  |
| BMI, kg/m^2^ | 0 Weeks | 31.3 (0.8) | 30.9 (0.9) | group: 0.454  time: <.001  group*time: 0.005 |
|  | 12 Weeks | 29.0 (0.8) | 27.7 (0.9) |  |
|  | 36 Weeks | 29.6 (0.9) | 28.7 (0.9) |  |
| WC, cm | 0 Weeks | 103.5 (1.9) | 102.2 (2.0) | group: 0.3147  time: <.0001  group*time: 0.152 |
|  | 12 Weeks | 94.4 (2.0) | 90.2 (2.1) |  |
|  | 36 Weeks | 95.9 (2.1) | 93.0 (2.2) |  |
| SBP, mmHg | 0 Weeks | 133.6 (2.8) | 126.7 (3.0) | group: 0.092  time: <.001  group*time: 0.662 |
|  | 12 Weeks | 120.2 (2.4) | 114.6 (2.6) |  |
|  | 36 Weeks | 119.4 (2.4) | 115.6 (2.5) |  |
| DBP, mmHg | 0 Weeks | 88.0 (2.5) | 84.6 (2.6) | group: 0.129  time: <.001  group*time: 0.883 |
|  | 12 Weeks | 77.0 (2.0) | 73.0 (2.1) |  |
|  | 36 Weeks | 78.6 (1.9) | 73.7 (2.0) |  |
| Glucose, mg/dl | 0 Weeks | 105.0 (9.3) | 118.2 (9.9) | group: 0.365  time: 0.303  group*time: 0.629 |
|  | 12 Weeks | 103.1 (2.2) | 104.1 (2.3) |  |
|  | 36 Weeks | 105.0 (3.5) | 106.9 (3.7) |  |
| TC, mg/dl | 0 Weeks | 199.3 (6.8) | 197.5 (7.2) | group: 0.275  time: <.001  group*time: 0.183 |
|  | 12 Weeks | 181.8 (6.1) | 170.1 (6.5) |  |
|  | 36 Weeks | 194.9 (6.9) | 180.7 (7.3) |  |
| Triglycerides, mg/dl | 0 Weeks | 150.5 (12.7) | 124.5 (13.5) | group: 0.287  time: 0.000  group*time: 0.309 |
|  | 12 Weeks | 113.6 (10.1) | 108.3 (10.7) |  |
|  | 36 Weeks | 142.8 (12.6) | 125.2 (13.3) |  |
| HDL-C, mg/dl | 0 Weeks | 51.7 (2.2) | 55.6 (2.3) | group: 0.315  time: <.001  group*time: 0.025 |
|  | 12 Weeks | 49.5 (1.9) | 49.2 (2.0) |  |
|  | 36 Weeks | 50.9 (2.1) | 55.7 (2.2) |  |
| LDL-C, mg/dl | 0 Weeks | 126.4 (5.4) | 122.1 (5.7) | group: 0.188  time: <.001  group*time: 0.247 |
|  | 12 Weeks | 115.0 (4.8) | 106.3 (5.1) |  |
|  | 36 Weeks | 115.3 (6.6) | 99.8 (7.0) |  |

BMI, body mass index; DBP, diastolic blood pressure; HDL-C, high-density lipoprotein cholesterol; LDL-C, low-density lipoprotein cholesterol; SBP, systolic blood pressure; SE, standard error; TC, total cholesterol; WC, waist circumference.

^a^Calculated using the linear mixed model.

**Appendix Table 3.** **Changes of individual sterols and their metabolic ratio from baseline through the follow-up period**

| Sterols, μg/mL | Time | Placebo + Phentermine | Orlistat + Phentermine | Overall p-value^a^ |
| --- | --- | --- | --- | --- |
|  |  | Estimated means (SE) | Estimated means (SE) |  |
| Cholesterol | 0 Weeks | 643.501 (25.307) | 653.759 (26.842) | group: 0.5123  time: <.0001  group*time: 0.1002 |
|  | 12 Weeks | 641.772 (26.970) | 592.020 (28.606) |  |
|  | 36 Weeks | 721.476 (26.061) | 694.999 (27.642) |  |
| Sitosterol | 0 Weeks | 0.537 (0.069) | 0.651 (0.074) | group: 0.9087  time: <.0001  group*time: 0.0936 |
|  | 12 Weeks | 0.450 (0.034) | 0.361 (0.037) |  |
|  | 36 Weeks | 0.636 (0.053) | 0.631 (0.056) |  |
| Campesterol | 0 Weeks | 0.810 (0.103) | 0.889 (0.109) | group: 0.5458  time: <.0001  group*time: 0.1454 |
|  | 12 Weeks | 0.602 (0.048) | 0.486 (0.050) |  |
|  | 36 Weeks | 0.914 (0.064) | 0.784 (0.068) |  |
| Stigmasterol | 0 Weeks | 0.125 (0.004) | 0.129 (0.004) | group: 0.5254  time: <.0001  group*time: 0.2618 |
|  | 12 Weeks | 0.101 (0.004) | 0.092 (0.004) |  |
|  | 36 Weeks | 0.121 (0.005) | 0.119 (0.006) |  |
| Chol_M | 0 Weeks | 37.080 (4.817) | 41.923 (5.109) | group: 0.3354  time: <.0001  group*time: 0.1928 |
|  | 12 Weeks | 96.363 (13.671) | 85.190 (14.501) |  |
|  | 36 Weeks | 247.435 (21.558) | 212.310 (22.866) |  |
| Chol_P | 0 Weeks | 380.090 (23.073) | 399.854 (24.473) | group: 0.7683  time: <.0001  group*time: 0.5258 |
|  | 12 Weeks | 532.260 (41.251) | 494.072 (43.753) |  |
|  | 36 Weeks | 846.647 (66.516) | 822.368 (70.551) |  |
| Chol_A | 0 Weeks | 328.974 (34.814) | 349.343 (36.926) | group: 0.1635  time: <.0001  group*time: 0.1573 |
|  | 12 Weeks | 1138.243 (137.810) | 845.890 (146.169) |  |
|  | 36 Weeks | 1700.619 (127.346) | 1569.177 (135.071) |  |
| Desmosterol | 0 Weeks | 62.949 (2.639) | 60.421 (2.799) | group: 0.2103  time: <.0001  group*time: 0.6219 |
|  | 12 Weeks | 54.379 (2.556) | 50.114 (2.711) |  |
|  | 36 Weeks | 68.702 (3.554) | 62.533 (3.770) |  |
| DHC | 0 Weeks | 84.859 (4.436) | 81.957 (4.705) | group: 0.3341  time: 0.0014  group*time: 0.8957 |
|  | 12 Weeks | 76.302 (3.175) | 72.648 (3.367) |  |
|  | 36 Weeks | 88.745 (4.072) | 82.886 (4.319) |  |
| Lathosterol | 0 Weeks | 461.457 (45.051) | 393.188 (47.783) | group: 0.1757  time: 0.0028  group*time: 0.5356 |
|  | 12 Weeks | 368.086 (39.632) | 308.793 (42.036) |  |
|  | 36 Weeks | 478.484 (53.520) | 360.565 (56.766) |  |
| Lanosterol | 0 Weeks | 83.994 (9.688) | 84.450 (10.276) | group: 0.3277  time: 0.0394  group*time: 0.1339 |
|  | 12 Weeks | 78.267 (8.895) | 68.160 (9.435) |  |
|  | 36 Weeks | 106.983 (14.647) | 74.482 (15.535) |  |
| 7α-OHC | 0 Weeks | 36.166 (7.465) | 57.367 (7.918) | group: 0.3645  time: <.0001  group*time: 0.0525 |
|  | 12 Weeks | 33.517 (3.757) | 28.312 (3.985) |  |
|  | 36 Weeks | 25.453 (2.115) | 23.146 (2.243) |  |
| 7β-OHC | 0 Weeks | 17.069 (0.971) | 20.191 (1.029) | group: 0.2773  time: <.0001  group*time: 0.0335 |
|  | 12 Weeks | 16.617 (0.700) | 16.010 (0.742) |  |
|  | 36 Weeks | 13.796 (0.318) | 13.715 (0.338) |  |
| Ketosterol | 0 Weeks | 23.210 (16.428) | 56.650 (17.424) | group: 0.1765  time: <.0001  group*time: 0.3694 |
|  | 12 Weeks | 17.636 (0.908) | 18.448 (0.963) |  |
|  | 36 Weeks | 12.244 (0.192) | 12.225 (0.203) |  |
| 27-OHC | 0 Weeks | 12.401 (0.575) | 12.635 (0.610) | group: 0.6495  time: 0.0004  group*time: 0.4693 |
|  | 12 Weeks | 12.955 (0.708) | 11.731 (0.751) |  |
|  | 36 Weeks | 14.518 (0.677) | 14.595 (0.718) |  |
| 24-OHC | 0 Weeks | 10.880 (0.928) | 10.828 (0.985) | group: 0.5201  time: <.0001  group*time: 0.2719 |
|  | 12 Weeks | 13.507 (1.409) | 10.686 (1.494) |  |
|  | 36 Weeks | 14.923 (0.823) | 15.691 (0.872) |  |
|  | 12 Weeks | 8.749 (0.454) | 8.293 (0.482) |  |
|  | 36 Weeks | 8.435 (0.371) | 8.634 (0.393) |  |
| Desmo_chol | 0 Weeks | 0.098 (0.003) | 0.094 (0.004) | group: 0.5228  time: 0.0139  group*time: 0.7422 |
|  | 12 Weeks | 0.087 (0.004) | 0.087 (0.004) |  |
|  | 36 Weeks | 0.096 (0.004) | 0.091 (0.004) |  |
| DHC_chol | 0 Weeks | 0.133 (0.006) | 0.127 (0.007) | group: 0.7935  time: 0.4204  group*time: 0.4962 |
|  | 12 Weeks | 0.122 (0.005) | 0.127 (0.006) |  |
|  | 36 Weeks | 0.125 (0.005) | 0.121 (0.006) |  |
| Latho/Chol | 0 Weeks | 705.672 (69.152) | 613.497 (73.347) | group: 0.2784  time: 0.0308  group*time: 0.605 |
|  | 12 Weeks | 571.774 (56.319) | 517.788 (59.735) |  |
|  | 36 Weeks | 647.224 (68.029) | 520.799 (72.155) |  |
| Lano/Chol | 0 Weeks | 126.229 (14.755) | 133.332 (15.650) | group: 0.6383  time: 0.3309  group*time: 0.1174 |
|  | 12 Weeks | 120.477 (13.136) | 118.153 (13.933) |  |
|  | 36 Weeks | 139.260 (16.429) | 107.123 (17.425) |  |
| Sito/Chol | 0 Weeks | 0.844 (0.099) | 0.991 (0.105) | group: 0.7343  time: <.0001  group*time: 0.1926 |
|  | 12 Weeks | 0.710 (0.063) | 0.640 (0.066) |  |
|  | 36 Weeks | 0.899 (0.081) | 0.922 (0.086) |  |
| Camp/Chol | 0 Weeks | 1.264 (0.150) | 1.361 (0.159) | group: 0.7706  time: <.0001  group*time: 0.2505 |
|  | 12 Weeks | 0.951 (0.090) | 0.858 (0.096) |  |
|  | 36 Weeks | 1.295 (0.106) | 1.159 (0.113) |  |
| Stigma/Chol | 0 Weeks | 0.201 (0.009) | 0.204 (0.009) | group: 0.8344  time: <.0001  group*time: 0.9753 |
|  | 12 Weeks | 0.164 (0.010) | 0.165 (0.011) |  |
|  | 36 Weeks | 0.172 (0.009) | 0.175 (0.010) |  |
| Latho/Camp | 0 Weeks | 764.434 (109.061) | 551.517 (115.677) | group: 0.3746  time: 0.0323  group*time: 0.1685 |
|  | 12 Weeks | 754.246 (105.985) | 717.372 (112.414) |  |
|  | 36 Weeks | 605.681 (73.962) | 520.056 (78.449) |  |
| 7α-OHC/Chol | 0 Weeks | 57.311 (9.543) | 85.761 (10.122) | group: 0.3319  time: <.0001  group*time: 0.0555 |
|  | 12 Weeks | 54.500 (6.242) | 47.920 (6.620) |  |
|  | 36 Weeks | 36.244 (3.039) | 33.656 (3.223) |  |
| 7β-OHC/Chol | 0 Weeks | 27.345 (1.375) | 31.255 (1.459) | group: 0.1927  time: <.0001  group*time: 0.1905 |
|  | 12 Weeks | 27.170 (1.490) | 27.845 (1.580) |  |
|  | 36 Weeks | 19.771 (0.700) | 20.143 (0.743) |  |
| 27-OHC/Chol | 0 Weeks | 19.767 (0.883) | 19.556 (0.936) | group: 0.7365  time: 0.2388  group*time: 0.6425 |
|  | 12 Weeks | 20.338 (1.144) | 20.468 (1.214) |  |
|  | 36 Weeks | 20.296 (0.909) | 21.443 (0.965) |  |
| 24-OHC/Chol | 0 Weeks | 17.236 (1.383) | 16.596 (1.467) | group: 0.6397  time: <.0001  group*time: 0.2728 |
|  | 12 Weeks | 20.944 (2.103) | 17.675 (2.231) |  |
|  | 36 Weeks | 21.141 (1.140) | 22.919 (1.209) |  |

7α-OHC, 7α-hydroxycholesterol; 7β-OHC, 7β-hydroxycholesterol; 24-OHC, 24-oxysterol; 27-OHC, 27-oxysterol; Chol, cholesterol; Chol-A, cholesteryl arachidonate; Chol-M, cholesteryl myristate; Chol-P, cholesteryl palmitate; Desmo, desmosterol; DHC, 7-dehydrocholesterol; Ketosterol, 7-ketocholesterol; Lano, lanosterol; Latho, lathosterol.

^a^Calculated using the linear mixed model.
